# CD11c+CD163+ Cells and Signal Transducer and Activator of Transcription 3 (STAT3) Expression Are Common in Melanoma Leptomeningeal Disease

**DOI:** 10.3389/fimmu.2021.745893

**Published:** 2021-10-08

**Authors:** Hinda Najem, Anantha Marisetty, Craig Horbinski, James Long, Jason T. Huse, Isabella C. Glitza Oliva, Sherise D. Ferguson, Priya U. Kumthekar, Derek A. Wainwright, Peiwen Chen, Maciej S. Lesniak, Jared K. Burks, Amy B. Heimberger

**Affiliations:** ^1^ Department of Neurological Surgery, Northwestern Medicine Malnati Brain Tumor Institute of the Lurie Comprehensive Cancer Center, Feinberg School of Medicine, Northwestern University, Chicago, IL, United States; ^2^ Department of Neurosurgery, Baylor College of Medicine, Houston, TX, United States; ^3^ Department of Pathology, Feinberg School of Medicine, Northwestern University, Chicago, IL, United States; ^4^ Department of Biostatistics, University of Texas M.D. Anderson Cancer Center, Houston, TX, United States; ^5^ Department of Pathology, University of Texas M.D. Anderson Cancer Center, Houston, TX, United States; ^6^ Department of Melanoma, University of Texas M.D. Anderson Cancer Center, Houston, TX, United States; ^7^ Department of Neurosurgery, University of Texas M.D. Anderson Cancer Center, Houston, TX, United States; ^8^ Department of Neuro-oncology, Feinberg School of Medicine, Northwestern University, Chicago, IL, United States; ^9^ Department of Leukemia, University of Texas M.D. Anderson Cancer Center, Houston, TX, United States

**Keywords:** melanoma, LMD, tumor microenvironment, T cells, dendritic cells, macrophages, STAT3

## Abstract

Leptomeningeal disease (LMD) in melanoma patients is associated with significant neurological sequela and has a dismal outcome, with survival measured typically in weeks. Despite the therapeutic benefit of targeted therapies and immunotherapies for Stage IV melanoma, patients with LMD do not typically benefit. A deeper understanding of the tumor microenvironment (TME) of LMD may provide more appropriate therapeutic selection. A retrospective analysis of subjects who underwent surgical resection with LMD (n=8) were profiled with seven color multiplex staining to evaluate the expression of the global immune suppressive hub - the signal transducer and activator of transcription 3 (STAT3) and for the presence of CD3+ T cells, CD68+ monocyte-derived cells, CD163+ immune suppressive macrophages, and CD11c+ cells [potential dendritic cells (DCs)] in association with the melanoma tumor marker S100B and DAPI for cellular nuclear identification. High-resolution cellular imaging and quantification was conducted using the Akoya Vectra Polaris. CD11c+ cells predominate in the TME (10% of total cells), along with immunosuppressive macrophages (2%). Another potential subset of DCs co-expressing CD11c+ and the CD163+ immunosuppressive marker is frequently present (8/8 of specimens, 8%). Occasional CD3+ T cells are identified, especially in the stroma of the tumor (p=0.039). pSTAT3 nuclear expression is heterogeneous in the various immune cell populations. Occasional immune cluster interactions can be seen in the stroma and on the edge. In conclusion, the TME of LMD is largely devoid of CD3+ T cells but is enriched in immune suppression and innate immunity.

## Introduction

Metastasis to the central nervous system (CNS) remains one of the most common and devastating complications of advanced melanoma. Up to 60% of metastatic melanoma patients are clinically diagnosed with CNS disease (1), which is a frequent site of treatment failure among patients treated with approved targeted and immune therapies. Among melanoma patients with CNS metastases, those with LMD have the worst outcomes, with overall survival from diagnosis of typically weeks to a few months (1, 2). Patients with parenchymal brain metastases have multiple treatment options including surgery, stereotactic radiosurgery, systemic therapies including the BRAF and MEK inhibitors, monotherapy with the immune checkpoint inhibitors ipilimumab or pembrolizumab, and/or the combination ipilimumab and nivolumab, which have demonstrated efficacy in clinical trials for metastatic melanoma patients with parenchymal brain metastases (3–8). In contrast, there are very few treatment options for LMD patients (9, 10), especially once patients develop LMD while on these agents. Notably, LMD often causes severe neurological deficits that can critically impact quality of life. Currently, the National Comprehensive Cancer Network guidelines recommend palliative radiotherapy and best supportive care for metastatic melanoma patients with LMD. As the incidence of LMD is rising, the focus on developing more effective treatments for LMD addresses an unmet clinical need of high clinical significance.

A major limitation for the use of immune checkpoint inhibitors or other types of modulators of tumor-mediated immune suppression for CNS malignancies is the low pre-existing immune T cell infiltration (11). Monotherapies directed at inhibition of operational immune suppressive mechanisms (i.e., maintenance of an effector response) would not be expected to exert significant therapeutic effects in the setting of CNS malignancies that have a paucity of T cells. Combinations of immunomodulatory agents targeting several modalities of the immune systems *in vivo* have the potential to tip the balance of immunosuppressive and immune-stimulatory signals within the tumor microenvironment to favor immune activation and targeted clearance of tumor. In melanoma, abundant innate immune infiltration of the TME (12) and the activation of STAT3 (13) has been shown to play a role in both tumorigenesis and the generation of immune suppression. Specifically, STAT3 expression in macrophages limits their activation (14) and inflammatory responses (15). When STAT3 is expressed within DCs, there is decreased maturation and decreased expression of MHC II, CD80, CD86, and IL-12, rendering them unable to stimulate T cells and to generate antitumor immunity (16). Expression of STAT3 in T cells abrogates their effector functions and induces Tregs (17). The immune composition of melanoma LMD throughout the tumor microenvironment has not been evaluated before, and current single cell techniques such as flow cytometry, single cell sequencing and CyTOF disrupt the tumor architecture and are deconstructive strategies that fail to provide tissue integration and spatial analysis. Therefore, to understand the immune modulation and composition of melanoma LMD more deeply, we conducted a retrospective study of human tissues that focus on observing the immune composition and profile created in the leptomeningeal space in melanoma LMD through seven color multiplex staining technique and high-resolution imaging.

## Materials and Methods

### Study Approval

Under an IRB-approved protocol, we identified patients that underwent surgical resection of melanoma metastasis to the leptomeningeal space from 1994 till 2019. All patients were screened based on a radiographic diagnosis of CNS leptomeningeal disease. Patient demographics and clinicopathological findings were collected from the electronic medical record (**Table 1**).

**Table 1:** Demographics of melanoma patients with LMD.

Age	Sex	Diagnosis	Location in the CNS	Pre-Op treatment
57	F	Melanoma/LMD	Cerebellum	Dabrafenib, Trametinib
29	M	Melanoma/LMD	Cerebellum	Chemotherapy
38	F	Melanoma/LMD	Occipital lobe	None
74	F	Melanoma/LMD	Temporal lobe	None
63	F	Melanoma/LMD	Cerebellum	Chemotherapy, IL-2, Ipilimumab
62	M	Melanoma/LMD	Occipital Lobe	Radiosurgery, Ipilimumab, Nivolumab
45	M	Melanoma/LMD	Temporal lobe	Allogeneic vaccine, whole brain radiation, chemotherapy
57	M	Melanoma/LMD	Frontal Lobe	Radiosurgery, Pembrolizumab, Dabrafenib

### Tissue Selection

Melanoma resections from the CNS that could have included the adjacent leptomeningeal space in subjects who had radiographic evidence of LMD were obtained from subjects ≥ 18 years old. Surgery indications in these patients is rare and is based on either establishing the diagnosis or for the alleviation of neurological symptoms thereby limiting the number of specimens available.

### Tissue Processing and Orientation

The tissue extracted by the surgeon was immediately fixed in 10% formalin and embedded in paraffin. Slides are created at 4 micrometers of thickness.

### Conventional Immunohistochemistry (IHC)

Each antibody was validated with conventional IHC before being incorporated into the multiplex panel. This technique consists of applying the primary antibody with its correspondent polymer in conjunction with DAB color development and hematoxylin stain. Preparation and rehydration of formalin-fixated paraffin-embedded tissue slides are done by incubation in a series of xylene (3x), 100% (2x), 95% (2x) and 75% ethanol (2x) solutions for 5 min each. Antigen retrieval is then performed by heating the slides at 120°C for 12 min while incubated in a specific antigen retrieval buffer (pH6 or pH 9, depending on the antibody used). Afterwards, the slides are washed and incubated in hydrogen peroxide quenching solution (10% methanol, 3% H_2_O_2_, and 87% pure water) for 15 min at room temperature (RT). A protein blocker is applied to the slides for 15 min at RT before applying the corresponding primary antibody. Final processing consists of applying the primary antibody specific polymer, coloring, and scanning.

### Multiplex Immunohistochemistry Staining

Each antibody was validated using conventional immunohistochemistry and monoplex immunofluorescence staining in conjunction with the corresponding opal fluorophore and the spectral DAPI counterstain. The antibodies were tested at three different dilutions, starting with manufacturer recommended dilution (MRD), MRD/2, and MRD/4 with 1/100 tyramide to select the optimal concentration of antibody capable of generating the best signal. The signal was then optimized through different tyramide titrations until generation of the best exposure time ranging from 5 to 25ms. Reproducibility was evaluated using: a positive control of each antibody with DAPI and a DAPI-alone slide. The negative controls included one unstained slide for auto-fluorescence compensation, a tyramide-alone slide treated with hydrogen peroxide for endogenous peroxidase masking, and finally a secondary antibody with tyramide slide. The following antibodies were used in the multiplex panel analysis: S100B Leica NCL-L-S100167 1/100 pH6 with Tyr480 1/200; pSTAT3 Cell Signaling Tyr705 D3A7 XP 1/200 pH 6 with Tyr520 1:150; CD68 Agilent PG-M1 1:50 pH 9 with Tyr570 1:150; CD3 Agilent clone F7.2.38 1:50 with pH 6 buffer for antigen retrieval with Tyr620 1:300; CD163 Abcam EPR19518 1:500 pH 9 with Tyr690 1:100; and CD11c Abcam EP1347Y 1:300 pH 9 with Tyr780 1:100.

### Image Processing

Slides were scanned with the Vectra Polaris Imaging System (Akoya) following the manual’s instructions using the Fluorescent Mode with high power field scan (40x). The microscope captures the multispectral fluorescent spectra, separately, at the corresponding tyramide Opal fluorophore wavelength with empirically determined exposure times. These captures are then stacked in one image without disrupting the unique fluorescent spectral signature of the markers. Phenochart software was used to visualize the images and stamp them. Finally, spectrally unmixed component images were reconstructed and exported for further analysis using Visiopharm software.

### Image Analysis and Quantification

All digitized unmixed images were analyzed using the Visiopharm software platform (Hørsholm, Denmark). Regions of interest (ROI) (e.g., tumor, stroma/infiltrative edge) were identified by a board-certified pathologist and transferred to the Visiopharm platform. To analyze cells within the different compartments, a series of customized algorithms were applied to all images and ROI. First, in order to exclude sample regions in which there was excessive bleeding, we trained a deep learning classifier (U-Net architecture) to identify these areas and exclude them from further analysis. Next, for the remaining tissue areas, we used a second Deep Learning algorithm (U-Net architecture) to identify DAPI positive nuclei and then expanded that boundary to approximate the true borders of the cell. The cell segmentation was confirmed *via* visual inspection using the membrane labels in the staining panel. Once cellular boundaries were confirmed, we used a directed approach to generate the targeted list of biomarker combinations in the samples: CD3, CD68, CD11c, and CD163 individual markers, dual CD68 CD11c, CD68 CD163, CD11c CD163, and triple CD68 CD11c CD163 combinations. For a given cell, the classification of each biomarker was gated using two independently controlled parameters: signal intensity and percent coverage. During the development of the classification algorithm, biomarker classification was visually inspected and, if needed, classification parameters were adjusted to maximize accuracy and minimize the number of false positives and false negatives for each biomarker. Once the parameters were optimized, the batch analysis cascade was applied to all images in the dataset. The results obtained were finally received and saved in Excel sheet format document.

### Statistical Analysis

For each cell marker or combined markers, a paired Wilcoxon test was performed to test whether there is difference in the percentages of cell populations between tumor area and stroma. Furthermore, in each area (tumor and stroma) another comparison using the same test was performed for each cell marker or combined markers depending on the expression of pSTAT3. At the end, p-values were obtained.

## Results

### Highlighted Immune Findings in Melanoma LMD

**Figure 1:**
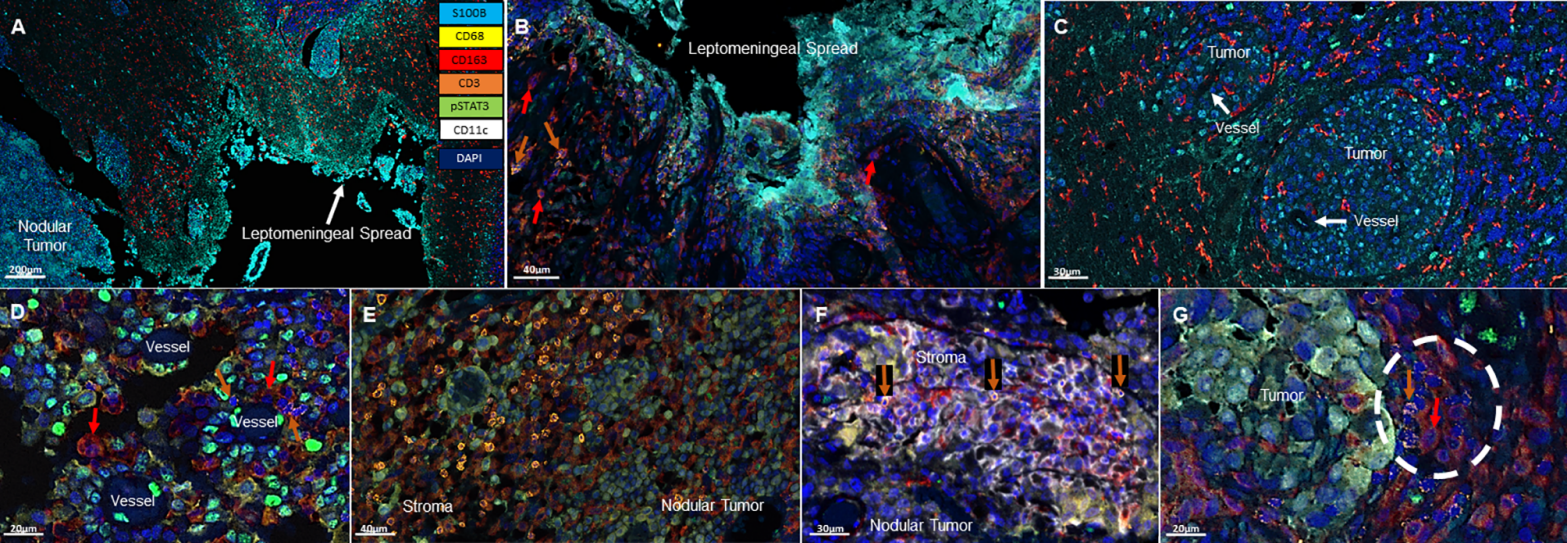
Representative immunofluorescent multiplex findings in melanoma LMD. **(A)** Gross findings of nodular tumor that was resected adjacent to the region of leptomeningeal spread. The specimens were stained with 7-color opal multiplex staining technique consisting of: Cyan-S100B tumor, Yellow-CD68, Red-CD163, Orange-CD3, Green-pSTAT3, White-CD11c and dark Blue-DAPI. **(B)** Higher magnified view of another image of LMD that displays CD163+ macrophages (red arrows) and occasional CD3+ T cells (orange arrows). **(C)** Perivascular cuffing of melanoma cells demonstrating abundant CD163+ macrophage infiltration. **(D)** In nodular melanoma, CD163+ macrophages (red arrows) and CD3+ T cells (orange arrows) are visualized in the perivascular areas. **(E)** CD3+ T cell infiltration in the stroma of nodular melanoma. **(F)** CD11c+ and CD3+ (orange arrows) cells cluster in the stroma of nodular melanoma. **(G)** CD163+ (red arrow) and CD3+ (orange arrow) cluster seen at the interface of the melanoma and the brain.

All specimens included a nodular LMD tumor which was the surgical intent of the resection and 3/8 of these specimens also included the distinctive leptomeningeal spread along the meninges (**Figure 1A**). Associated with the leptomeningeal component were surrounding immunosuppressive CD163+ macrophages (3/3) (**Figure 1B**). Occasional T cells could be identified (2/3) in proximity to the leptomeningeal spread. In the areas of tumor perivascular cuffing, CD163+ single cells were the dominant immune population (**Figure 1C**). No CD11c+ single cells are noted in these regions.

**Figure 2:**
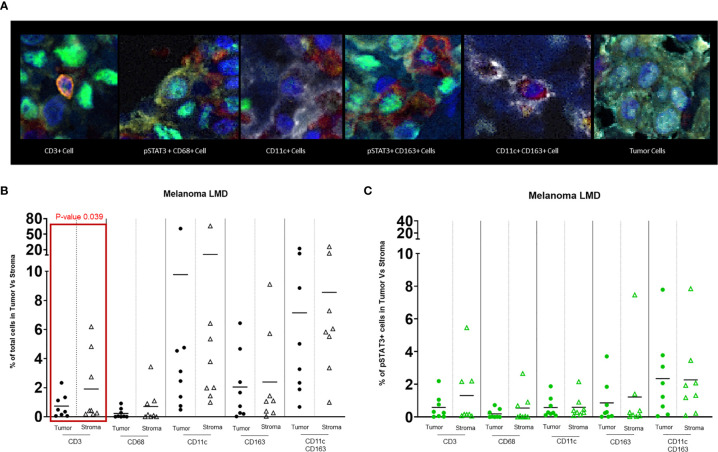
**(A)** Representative images of different cell populations seen in the TME of melanoma LMD: pSTAT3-CD3+ T cells, pSTAT3+CD68+ macrophages, pSTAT3-CD11c+ APCs, pSTAT3+CD163+ macrophages, pSTAT3-CD11c+CD163+ APCs, and tumor cells demonstrating heterogenous nuclear expression of pSTAT3. **(B)** Dot-plot representing the percentages of total immune cells in the tumor area vs stroma. The black circles represent the total immune cell populations in the tumor and the black-outlined triangles represent the total immune cells in the stroma. Each circle or triangle represent a specimen. There is a significant difference between the percentages of CD3+ T cells in the stroma vs in the tumor (p= 0.039). **(C)** Dot-plot representing percentages of pSTAT3+ immune cells in the tumor area vs stroma. The green circles represent pSTAT3+ immune cell populations in the tumor area and the green-outlined triangles represent pSTAT3+ immune cells in the stroma. Each circle or triangle represent a specimen.

In the nodular regions of the melanoma, the tumor vasculature demonstrated immune infiltration of both T cells and macrophages (**Figure 1D**). T cells were usually seen in the tumor stroma (**Figure 1E**). In comparison to the immune populations in the regions of leptomeningeal spread, the immune microenvironment in the melanoma nodule were enriched with CD11c+ single cells seen in the tumor and the stroma. Dual co-expressing CD11c+ and CD163+ cells were also present in the TME. Occasional interactions between CD11c+ single cells and T cells (4/8) could be seen in the tumor stroma (**Figure 1F**). Interactions of CD163+ macrophages and T cells were identified at the edge of the tumor and the brain (3/8) (**Figure 1G**). In general, these cluster interactions occurred between cells that lacked expression of pSTAT3, although pSTAT3 expression was a frequent finding in all the specimens (8/8) (**Figure 2A**).

### T Cells Partition to the Tumor Stroma

**Figure 3:**
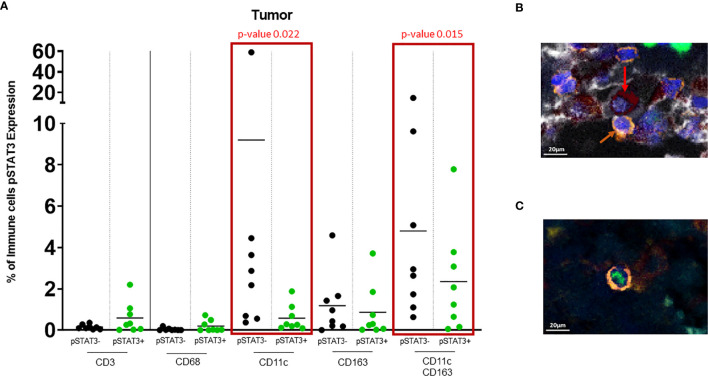
**(A)** Dot plot showing % of pSTAT3- vs pSTAT3+ cells in the tumor area. Green circles represent pSTAT3+ populations and black circles represent pSTAT3- populations. Each circle represents a specimen. Red-outlined rectangles highlight the significative comparisons with p-values ≤ 0.05. CD11c+pSTAT3- cells and dual CD11c+CD163+pSTAT3- cells are significantly higher relative to CD11c+pSTAT3+ cells (p=0.022) and CD11c+CD163+pSTAT3+ cells (p=0.015) respectively. The other immune cell populations do not show any significant difference. **(B)** In tumor stroma, pSTAT3- T cells (orange arrows) can be seen in tight proximity to CD11c+CD163+pSTAT3- cells (red arrows). **(C)** pSTAT3+ T cell isolated in tumor.

CD3+ T cells typically localize to the stroma within the tumor (p=0.039) (**Figure 2B**). Despite no significant differences in the frequency of pSTAT3 expressing immune cells in regions of tumor relative to the stroma (**Figure 2C**), there was a higher frequency of pSTAT3+ T cells in the tumor (**Figure 3**).

### CD11c+ Cells Are Enriched in LMD Disease

There was robust infiltration of melanoma with CD11c+ expressing cells (CD11c+ single cells and dual CD11c+CD163+ cells (**Figure 2B**), representing a mean of 10% and 8% of total cells, respectively, that lack pSTAT3 expression (**Figure 3A**), indicating antigen presentation capacity. Nonetheless, there was also a substantial population of CD11c+pSTAT3+ and co-expressing CD11c+CD163+pSTAT3+ cells, suggesting the presence of immune suppression/dysfunction.

In 4/8 of the specimens, especially in the stroma, T cells were observed in close proximity to dual CD11c+CD163+ cells that lacked pSTAT3 expression reflective of antigen presentation capacity to T cells (**Figure 3B**). In contrast, pSTAT3+ expressing immune cells were generally seen isolated within the tumor and stroma (**Figure 3C**).

### CD163+ Macrophages Are Frequent in LMD

CD163+ macrophages diffusely infiltrate tumor and stroma with no significant difference between the different areas (mean of 2% between all specimens in the tumor and stroma) (**Figure 2B**). No difference was detected between the frequency of pSTAT3+ macrophage populations in the tumor and the stroma (**Figure 2C**) and there was heterogeneity of pSTAT3 expression within macrophages in the different areas of the tumor similar for T cells and CD11c+ cells.

## Discussion

This study describes the immune composition within melanoma metastasis to the leptomeningeal space using high resolution cell imaging. As mentioned before, this is a rare oncological population for analysis, since most patients rapidly succumb to their disease, and surgical resection is rarely indicated. Our study subjects had radiographically confirmed LMD and underwent surgical resection for an adjacent mass secondary to neurological symptoms or to determine a diagnosis. In order to evaluate potential immune therapeutic strategies that might be rationally selected, a basic understanding of the immune composition of LMD must be the first step. STAT3 has been shown to play a critical role in melanoma brain metastasis (18). Our study indicates that this is an operational mechanism in the setting of LMD in a wide variety of infiltrating immune cells. As such, blood-brain-barrier penetrant inhibitors that are in clinical trials, such as WP1066 (NCT01904123), may be a viable therapeutic strategy. In contrast, T cell infiltration was an uncommon event in this setting, and these cells were usually confined to areas of bulky tumor stroma. Given this fact, strategies like STING agonists that generate tumor infiltration of T cells into the TME may need to be considered if an immune checkpoint inhibitor therapy approach is being employed. Similar to gliomas, LMD in melanoma patients appears to demonstrate an enrichment of innate immune cells like CD68+ single cells (monocyte-derived cells) and CD163+ single cells or macrophages. The predominant CD163 expression in these innate immune cells suggest polarization towards immune suppression; therapeutic strategies that polarize these cells to a more favorable pro-inflammatory phenotype such as CSF-1R inhibitors (19), STING agonists (20), and/or anti-PD-1 (21), could therefore be considered in this context. CSF1 inhibitors may further enhance the anti-tumor efficacy of BRAF inhibition in preclinical models (22) and could also be considered.

CD11c+ single cells are present in the TME of LMD but many of those CD11c+ cells also demonstrated co-expression of CD163. We postulate that these cells might not be immune suppressive but rather inflammatory DCs, where they have been described in association with CD3+ T cells during lupus nephritis (23). These dual CD11c+CD163+ expressing cells have also been identified in adipose tissues (24). They have been shown to positively correlate with T cell infiltration and prolonged survival in oropharyngeal cancers (25). Recently, Inna Smalley et al. showed that the presence of DC3 in the TME, which is a new subset of DCs expressing the CD163 marker among others (26), was associated with increased overall survival in patients with melanoma CNS metastases regardless of the tumor location and treatment (27). Despite the description of this unique immune cell population by others, the immunological functional consequence of this phenotype is unknown and may be contextual. DCs, especially the cDC1 subset, if present in the TME of brain tumors, may be an important contributor of antigen presentation and T cell activation (28) and can serve as a biomarker for a positive response to immunotherapies. In contrast, their absence is correlated to poor prognosis (29, 30). The functional consequences of these newly identified immune subset will need to be elucidated and indicates the current lineage characterization, especially in the context of cancer, is insufficient. With the burgeoning use of multiplex immunohistochemistry and mass cytometry, the identification of new immune states and phenotypes will likely requiring revision of designations based on immune functional characterization. Thus, subsets of DCs that have been described thus far may go well beyond the classical definitions of plasmacytoid DCs and myeloid cDC1 and cDC2. In several inflammatory diseases and cancers, the involvement of CD11c+ DCs has been shown to activate T cells and induce an effective immune response. If the DC-T cell interactions observed in the TME represents an early activation event of T cells that is happening within the TME before irreversible T cell exhaustion, reversing the hypothesis that T cells activation happens in the peripheral cervical lymph nodes, then the presence of this interactions may be a novel biomarker predictive of responses to immune checkpoint inhibitors.

In addition to the descriptive nature of our study, several additional limitations exist mostly involving the small number of subjects that impeded sufficient statistical power to draw meaningful conclusions regarding specific immunological findings relative to patient outcomes. In addition, we acknowledge that using only seven markers is insufficient to completely characterize the different immune cell phenotypes of the TME and thus additional complementary identification analysis are needed. Correlation of the association of various immune cells with a validation dataset from the Cancer Genome Atlas would have been beneficial, but unfortunately, melanoma LMD cases are not available. Tissue segmentation also posed technical challenges since identifying specimens to interrogate the tumor/CNS interface was limited due to tissue banking protocols that tended to enrich for specimens that had larger amounts of relatively pure tumor, and fewer specimens containing adjacent normal tissues. Nonetheless, this characterization provides insights into applicable therapeutic strategies, although it will need additional interrogation of the functional immunological consequences of the newly identified subsets.

## Data Availability Statement

The original contributions presented in the study are included in the article/supplementary material. Further inquiries can be directed to the corresponding author.

## Author Contributions

Conception and Design: SDF and ABH. Data analysis and interpretation: HN, AM, CH, JL, JTH, ICGO, PUK, DAW, PC, JKB, and ABH. Manuscript writing: HN and ABH. Accountable for all aspects of the work: All authors. All authors contributed to the article and approved the submitted version.

## Funding

The funding agencies including the NIH R01 NS120547 and Codiak BioSciences Inc had no role in the study design, collection, analysis, interpretation of data, the writing of this article or the decision to submit it for publication.

## Conflict of Interest

AH serves on the advisory boards of Caris Life Sciences and WCG Oncology, receives royalties on licensed intellectual property from Celldex Therapeutics and DNAtrix, and received research support from Codiak BioSciences Inc.

The remaining authors declare that the research was conducted in the absence of any commercial or financial relationships that could be construed as a potential conflict of interest.

## Publisher’s Note

All claims expressed in this article are solely those of the authors and do not necessarily represent those of their affiliated organizations, or those of the publisher, the editors and the reviewers. Any product that may be evaluated in this article, or claim that may be made by its manufacturer, is not guaranteed or endorsed by the publisher.
